# Nicotinamide mononucleotide enhances fracture healing by promoting skeletal stem cell proliferation

**DOI:** 10.7150/thno.98149

**Published:** 2024-09-16

**Authors:** Yitian Shi, Jiayin Peng, Mengfan Liu, Xiling Qi, Siyu Li, Qiangqiang Li, Qing Jiang, Liming Zheng, Jiankun Xu, Yun Zhao, Yifeng Zhang

**Affiliations:** 1 School of Life Science and Technology, ShanghaiTech University, Shanghai 201210, PR China.; 2 Shanghai Clinical Research and Trial Center, Shanghai 200000, PR China.; 3 Key Laboratory of Multi-Cell Systems, Shanghai Institute of Biochemistry and Cell Biology, Center for Excellence in Molecular Cell Science, Chinese Academy of Sciences; University of Chinese Academy of Sciences, Shanghai 200031, PR China.; 4 Division of Sports Medicine and Adult Reconstructive Surgery, Department of Orthopedic Surgery, Nanjing Drum Tower Hospital, Affiliated Hospital of Medical School, Nanjing University, 321 Zhongshan Road, Nanjing, Jiangsu 210008, PR China.; 5 Department of Orthopedic Surgery, the Second Affiliated Hospital, Zhejiang University School of Medicine, Hangzhou, Zhejiang 310000, PR China.; 6 Musculoskeletal Research Laboratory, Department of Orthopedics & Traumatology, The Chinese University of Hong Kong, Hong Kong 999077, PR China.

**Keywords:** fracture healing, callus formation, skeletal stem cells, NAD

## Abstract

The process of skeletal regeneration initiated by stem cells following injury, especially in fractures, is significantly impaired by aging and adverse factors. Nicotinamide mononucleotide (NMN), a critical endogenous precursor of nicotinamide adenine dinucleotide (NAD), has garnered extensive attention for its multifaceted regulatory functions in living organisms and its wide-ranging therapeutic potential. However, whether NMN contributes to trauma-induced skeletal regeneration remains unclear.

**Methods**: The transverse femoral shaft fracture model was employed to evaluate the potential advantages of NMN administration for overall repair during the initial fracture stages in male mice through micro-CT analysis, histochemistry, and biomechanical testing. The pro-proliferative function of NMN on skeletal stem cells (SSCs) was investigated through flow cytometry, qRT-PCR, NAD content measurement, and cell proliferation assay.

**Results**: In this study, we observed that the administration of NMN during the initial phase of fracture in mice led to a larger callus and corresponding improvement in micro-CT parameters. NMN enhances the cartilaginous component of the callus by elevating the NAD content, consequently accelerating subsequent endochondral ossification and the fracture healing process. Subsequent analyses elucidated that NMN was beneficial in promoting the expansion of diverse stem cells *in vivo* and *in vitro* potentially via modulation of the Notch signaling pathway. Moreover, the depletion of macrophages profoundly obstructs the proliferation of SSCs.

**Conclusion**: Our discoveries provide a potential strategy for enhancing fracture healing through stimulation of callus SSC proliferation at an early stage, shedding light on the translational value of NMN as an enhancer for skeletal regeneration and highlighting the pivotal role of macrophage-stem cell interactions in governing the regenerative influence of NMN on stem cells.

## Introduction

Bone regeneration following a fracture is characterized as a complex process dominated by endochondral osteogenesis, involving the sequential transition from a cartilaginous callus to woven bone with bone marrow cavity 1. Serving as an intermediate scaffold, the soft callus furnishes temporary support to stabilize the fractured bone and lay the foundation for the hard callus formation. It is a quintessential criterion for evaluating early fracture healing, judged by its callus size. Insufficient and a smaller cartilaginous callus caused by aging 2, diabetes 3 and smoking 4 can hinder subsequent new bone formation, leading to compromised healing outcomes. Conversely, a larger cartilaginous callus can promote fracture repair, as evidenced by treatments like administrated estrogen 5, magnesium implants 6, and teriparatide 7. Diverse stem cell populations are recruited to initiate proliferation at the fracture site leading to the subsequent formation of a cartilaginous callus, which is essential for overall fracture repair. To identify heterogeneous subpopulations within skeletal stem cells (SSCs), researchers have developed lineage-tracing mouse models. These models are designed to label cells expressing distinct markers, including Gli1, α-SMA, CTSK, and Grem1, which play crucial roles in embryonic development, bone maintenance and the regeneration process following injury 8-12. A breakthrough advancement was achieved by Chan and colleagues, who identified a specific population of SSCs in both mice and humans utilizing flow cytometry-based technology. These SSCs sit atop the hierarchical tree and have the capability to generate bone cartilage stroma progenitor cells (BCSPs), which further differentiate into bone, cartilage, or stromal cells 13-15. While stem cells are integral to fracture repair, elucidating the mechanisms that trigger their proliferation requires further exploration.

Nicotinamide mononucleotide (NMN), a pivotal biosynthetic precursor and booster of nicotinamide adenine dinucleotide (NAD), is essential for maintaining NAD homeostasis. It regulates NAD-dependent enzymes including the Sirtuins family and poly (ADP-ribose) polymerases, which are vital in mitochondrial metabolism, DNA damage repair and immune activation of T cells 16-18. In addition to its role in attenuating aging 19-21, NMN supplementation has shown therapeutic benefits in diseases linked to NAD metabolic disturbances, including heart failure 22, kidney injury 23 and neurodegenerative diseases 24. Notably, Zhang *et al.* discovered that nicotinamide riboside (NR) administration accelerates the regeneration of skeletal muscle by enhancing the function of skeletal muscle stem cells 25, highlighting the potential of NAD precursors in tissue repair mechanisms. Moreover, previous research indicates that the loss of bone mass and subsequent osteoporosis associated with aging can be mitigated through supplementation with NAD intermediates 26, 27. However, it remains unclear whether NMN contributes to bone fracture repair.

In this study, we investigated the effects of NMN on bone regeneration employing a transverse femoral shaft fracture model. Our findings suggest that supplementation of exogenous NMN enhanced cartilage callus formation by elevating the total NAD levels within the callus. Furthermore, we validated that the observed increase in cartilage components was attributed to the regeneration potential of heterogeneous SSCs activated by NMN. Additionally, NMN effectively enhanced the expansion of SSCs *in vitro*. Depletion of macrophages using chlorophosphate liposomes abolished the stimulatory effect of NMN on SSCs, highlighting the critical role of macrophage-stem cell interaction in initiating callus formation. Together, this finding underscores the relationship between NAD and the regenerative capability of stem cells during bone injury healing, thus paving the way for advancing NMN as a potential treatment for fractures and even osteoporosis.

## Methods

### Animals

All animal studies involving mice were approved by the Institutional Animal Care and Use Committee of ShanghaiTech University and performed in conformity with governmental regulations of China for the care and use of animals. C57BL/6J mice were purchased from GemPharmatech. Gli1-CreERT2; R26-tdT mice were injected intraperitoneally with 200 μg per gram body weight of 20 mg/ml Tamoxifen (dissolved in corn oil, T5648, Sigma) daily for 3 consecutive days. Unless stated otherwise, male mice aged 8 to 9 weeks were utilized.

### Mouse femoral fracture and dill hole injuries

Before surgery, mice were anesthetized via intraperitoneal injection of 2.5% avertin. To expose the femur, an incision was made along its anterior side in the open transverse femoral fracture model. A 25-gauge syringe needle was inserted into the intramedullary canal through the femoral platform, followed by creating a transverse fracture using a dental machine equipped with a 0.5 mm-diameter bistrique. Subsequently, the needle threaded through the proximal femur was bent to form a curved hook and re-threaded through the skin for fracture fixation and to enable post-surgery motion. The fractured bones were realigned, and the incision was closed using a 5-0 synthetic absorbable suture. Mice were equally divided into control and experimental groups, receiving intraperitoneal injections of saline, FK866 (10 mg/kg, Selleck), NMN (500 mg/kg, Bidepharm), and a combination of FK866 plus NMN after fracture. In cases of drill hole injuries, a 0.6-mm mono-cortical hole was created using a dynamoelectric dental drill (Seayong).

### Biomechanical testing

Uninjured and fractured femurs were harvested at 5 weeks post-fracture. Surrounding soft tissues were cleaned, and the intramedullary needle was removed. The biomechanical properties of the femur samples were evaluated until failure using a Universal Testing System with a 100N load cell (INSTRON). The load was continuously applied to the callus region at a 0.5 mm/min displacement rate until the femur broke, and the maximum load was recorded. Unfractured femurs were used as the control.

### Micro-CT analysis

Mice were anesthetized and immobilized at 7, 14, 21, 28, and 35 days post fracture. The fractured femur containing the intramedullary needle or bone-defected region was scanned using a high-resolution microCT scanner (Skyscan 1276; Bruker, Germany) with a source voltage of 60 kV, a source current of 200 µA, a filter setting of AI 0.5 mm, and a pixel size of 20 microns at 2016 × 1344 resolution. After data acquisition, reconstruction, and position correction were performed using Skyscan Nrecon software v2.0.0.5 and DataViewer v1.5.6.2, respectively. The prosthetic bone properties were measured using CT Analyser v1.17.7.2. Morphometric analysis of callus was conducted after excluding the fixed needles by defining the volume of interest. Three-dimensional reconstructions were performed using CTVox v3.3.0 for visualization.

### Histochemistry and immunofluorescence

Freshly dissected femurs were fixed in 4% paraformaldehyde at 4 °C for 7 hours and then decalcified in 8% EDTA for 2 weeks, with a weekly substitution of EDTA. After decalcification, part of the bones was dehydrated, embedded in paraffin, and then manually sectioned at a thickness of 10 µm for subsequent Movat's pentachrome staining. The remaining samples were successively dehydrated in 15% and 30% sucrose at 4 °C overnight before embedding in OCT. Representative 10 µm frozen sections were collected and stored at -20 °C. Tissue immunohistochemistry was performed using PCNA antibody (AF1363, Beyotime, 1:50) and CD31 antibody (ab222783, Abcam, 1:100).

### NAD quantification assay

NAD+ levels in BMSCs and fracture callus were measured using a commercially available NAD/NADH assay kit (Beyotime) according to the manufacturer's instructions. To detect NAD content in the fracture callus, each muscle-removed callus was weighed and homogenized in NAD extraction buffer, followed by centrifugation at 12,000 rcf for 10 min at 4 °C. The supernatant was then used in the enzymatic reactions to quantify NAD_total_ at 450 nm using a multimode plate reader (MD). Total NADH and NAD+ were quantified in BMSCs.

### Flow cytometry analysis

Various types of cells contributing to early callus formation including heterogeneous BMSCs and skeletal stem cell lineage, were isolated as previously described. In brief, fracture calluses were dissected, finely cut into pieces, and ground vertically with a mortar and pestle in a digestion buffer containing 2.2 mg/ml collagenase II (Yeasen), 1.6 mg/ml hyaluronidase (Coolaber), 100 U/ml DNase I (Roche), 1 mg/ml poloxamer 188 (Sigma-Aldrich), and 20 mM HEPES (Beyotime) in Hanks' Balanced Salt Solution with Ca^2+^ and Mg^2+^ (Beyotime). Bone fragments were enzymatically digested at 37 °C under agitation for 1 hour. The incubation was terminated by adding multiple volumes of cell-suspension buffer, which was then followed by passing through a 70 µm nylon mesh and centrifuged at 500 rcf at 4 °C for 5 min. Following lysis of red blood cells and removal of deposited bone debris by gravity, mononuclear cells were collected and blocked with TruStain FcX PLUS (Biolegend) for 10 min, followed by staining with combinations of the following antibodies: anti-CD45-PE (Biolegend), anti-Ter119-PE Biolegend), anti-Tie2 (Biolegend), anti-6C3-PE-CY7 (Biolegend), anti-Thy1.1-FITC (Thermo Fisher), anti-Thy1.2-FITC (Thermo Fisher), anti-CD51-Biotin (Biolegend), anti-CD105-APC (Biolegend), Streptavidin-AF680 (Bioss), anti-CD140a-FITC (Miltenyi), anti-CD140b-Biotin (Biolegend), anti-F4/80-FITC (Biolegend), anti-CD11b-PE-CY7 (Biolegend), anti-LEPR (Sino), anti-ACTA2 (ABclonal) and goat-anti-Rabbit IgG (H+L)-FITC (APExBIO). Cells were incubated with primary antibodies for 30 min, followed by incubation with secondary antibodies for an additional 30 min. DAPI staining was used to exclude dead cells. Flow cytometric analyses were performed using an LSRFortessa X20 (BD) and analyzed with FlowJo v10.6.2. Cell sorting was performed using the SORP ARIA Fusion (BD) instrument.

### Isolation of mesenchymal stem cells and bone marrow-derived macrophages

To isolate primary BMSCs and monocytes, bone marrow was flushed from the femurs and tibias of C57BL/6J mice using HBSS buffer to yield whole bone marrow cells. After washing and filtering, the bone marrow cells were seeded in a 10-mm dish. After 6 hours, the adherent cells and the supernatant containing non-adherent cells were collected separately. The adherent BMSCs were cultured in a growth medium (α-MEM medium supplemented with 10% FBS and 1% penicillin-streptomycin) at 37 °C with 5% CO2. Passage 1 to 2 BMSCs were used for migration, viability, quantitative RT-PCR, and NAD measurement experiments. Furthermore, the supernatant containing monocytes was cultured in a growth medium supplemented with 20 ng/ml M-CSF for 7 days. Macrophages were differentiated into M1 and M2 by stimulation with 100 ng/ml LPS (Invitrogen) and 10 ng/ml IL-4 (novoprotein), respectively.

### Macrophage transplantation

For intraoperative cell transplantation, bone marrow-derived monocytes were prepared and cultured in growth medium supplemented with 20 ng/ml M-CSF for 7 days to induce their differentiation into macrophages. Then, 1ⅹ10^6^ trypsinized macrophages were instilled into the femoral gap created during fracture surgery.

### CFU-F assay

Freshly flushed bone marrow plugs underwent enzymatic digestion using 2 mg/ml Collagenase II (Yeasen), 3 mg/ml Dispase II (Coolaber), and 100 U/ml DNase I (Roche) for 1 hour. The cells were then seeded in 6-well plates at a seeding density of 5x10^5^ cells per well and cultured in growth medium supplemented with 10 µM ROCK inhibitor Y-27632 (Aladdin). The culture medium was replaced every 3 to 4 days. Colonies were fixed in 4% paraformaldehyde and stained with Toluidine blue (Coolaber) for up to 12 days after plating, and then stained colonies were counted. Additionally, 600 FACS-sorted mouse SSCs and BCSPs were seeded in gelatin-coated 6-well plates and cultured for 14 days.

### Cell viability assay

Cell viability was measured using the Cell Counting Kit-8 (share-bio). Primary BMSCs or BMDMs in the logarithmic growth phase were seeded into 96-well cell culture plates at a density of 1×10^4^ per well and incubated at 37 °C with 5% CO_2_ for 24 hours. Subsequently, the cells were treated with varying concentrations of FK866 (Selleck, 0-60 nM) or NMN (Bidepharm, 0-100 µM) for 24 hours before adding of 10 µL of CCK-8 reagent. After further incubation for 1 or 2 hours at 37 °C, the optical density at 450 nm was measured using a multimode plate reader (MD).

### Scratch wound assay

P2 BMSCs were seeded in a 6-well plate and cultured in a growth medium till reaching 80-90% confluence. A 200 µL pipette tip was then used to create an approximately 0.5 mm scratch, following which the cells were rinsed with PBS to remove cellular debris. Subsequently, BMSCs were allowed to migrate with or without NMN (10 µM) in α-MEM medium supplemented with 1% FBS for 48 hours. The migration of BMSCs was photographed at the marked location using an optical microscope (Olympus) at 0, 24, and 48 hours, and the width of the gap was measured using Fiji software.

### Chondrogenic differentiation

For chondrogenic differentiation, FACS purified 75000 SSCs and 50000 BCSPs in 5 µL basal medium were seeded in a 12-well plate, and followed by adhesion at 37 °C for 2 hours. Besides, trypsinized 10^5^ C3H10T1/2 cells were plated in a 12-well plate as in micromass or monolayer cultures. Chondrogenic medium corresponds to DMEM with 10%FBS, 100 nM dexamethasone, 1 µM L -ascorbic acid and 10 ng/ml TGFβ1 (novoprotein) was added and replenished every 3 days. After 20 days, the cartilage matrix was fixed in 4% paraformaldehyde and stained with alcian blue (Solarbio).

### Cell proliferation assay

To assess the effects of NMN on Gli1^+^ periosteal cell proliferation within the callus, EdU was intraperitoneally injected at a dose of 500 µg per mouse at 3 and 5 days post-surgery. Fractured femurs were harvested at DPF7 for bone sectioning, and frozen sections were stained for EdU using the Click-iT EdU imaging kit (Invitrogen). For the EdU proliferation assay *in vitro* using C3H10T1/2 cell lines, an EdU cell proliferation kit (Beyotime) was utilized.

The proliferative capacity of C3H10T1/2 cell lines was assessed by seeding cells in 6-well plates at a density of 1×10^5^ cells per well and culturing them in a growth medium for 50 hours. The population doubling time (PDT) was calculated using the following formula: PDT = t × [lg2/(lgNt - lgN0)], where t is the culture period, N0 is the initial seeded cell number, and Nt is the harvested cell number.

### RNA extraction and quantitative RT-PCR

SSCs, BCSPs, and macrophages isolated from fractured calluses were FACS sorted, followed by total RNA extraction using the Hipure Total RNA Nano Kit (Magen) according to the manufacturer's instructions. RNA was extracted from both cultured cells and freshly dissected tissue using the EZ-press RNA Purification Kit (EZBioscience). The cDNA was prepared using the HiScript II Q Select RT SuperMix for qPCR with gDNA wiper (Vazyme) and used for quantitative RT-PCR performed with the ChamQ Universal SYBR qPCR Master Mix (Vazyme). Transcript expression levels were calculated using the 2^-ΔΔCt^ method, with the reference gene β-actin used to normalize variability between samples. A list of primers is shown in Table S1.

### Western blot analyses

Bone proteins were extracted from fractured callus using RIPA buffer supplemented with PMSF (Beyotime). SDS-PAGE separated the proteins, transferred them onto a nitrocellulose membrane, and probed with specific antibodies against Hey1 (19929, Proteintech), Hes1 (A0925, ABclonal), and an anti-Vinculin antibody (A2752, ABclonal) as an internal standard.

### shRNA interference

Lentiviral vectors, including pLKO.1-Nampt (shNampt), pLKO.1-Scramble (shNC), pLENTI-Nampt (AdNampt), pLENTI-Nampt-GFP (AdNampt-GFP), and pLENTI-NC (AdNC), were constructed and co-transfected into HEK293 cells with psPAX2 and Pmd2G using Lipofectamine 3000 reagent, following the manufacturer's instructions. Viral particles were collected and used to transfect the C3H10T1/2 cells.

### Statistical analysis

Two-tailed Student's t-tests were utilized to compare the two groups. For unpaired comparisons involving more than two groups, one-way analysis of variance (ANOVA) followed by Tukey's multiple comparison tests was employed. A standard two-way ANOVA was additionally employed to compare the paired data at different time points. Statistical analyses were conducted using GraphPad Prism 9 (GraphPad). Deviations are presented as the standard error of the mean. A P-value of less than 0.05 (*p* < 0.05) is considered statistically significant. Unless otherwise specified in the legend, an asterisk (*) denotes a P-value of less than 0.05.

## Results

### Exogenous NMN supplementation plays a beneficial role in the repair of bone injuries

**Figure 1:**
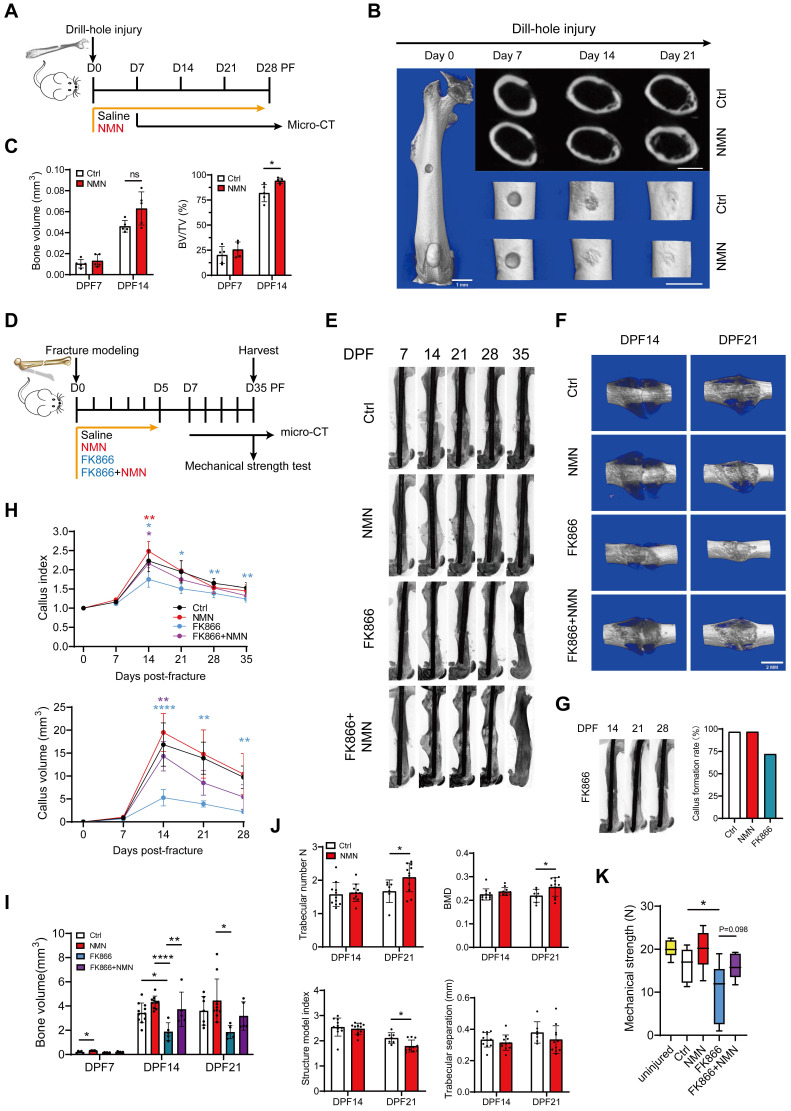
** NMN promotes bone defects and fracture repair in adult mice.** (A) Schematic of NMN administration after drill-hole defect creation and following assessment by micro-CT. (B) Representative micro-CT 2D and 3D images showing the healing of the defective femur in control and NMN-treated mice. Scale bar: 1 mm. (C) Micro-CT analysis of bone volume and bone volume/total volume in the defect region (n = 5). (D) Schematic illustrating the administration of Saline, NMN, FK866, and a combination of FK866 and NMN after fracture creation, followed by assessments. (E) Representative radiographs of injured femurs were taken weekly from day 7 to day 35 post-operation. (F) Micro-CT 3D images showing the callus. (G) Micro-CT images of fractured femurs from the FK866 group reveal the absence of callus formation during the mid to late stages of fracture healing. The percentage of callus formation differs between the administered groups. (H) The callus index and callus volume curves were used to assess differences in callus size among the treatment groups at various time points post-fracture (For the callus index: Ctrl group had n = 6 at day 7, n = 11 at day 14, n = 7 at day 21, n = 7 at day 28, and n = 7 at day 35; NMN group had n = 6 at day 7, n = 11 at day 14, n = 12 at day 21, n = 10 at day 28, and n = 11 at day 35; FK866 group had n = 5 at day 7, n = 6 at day 14, n = 6 at day 21, n = 6 at day 28, and n = 6 at day 35; FK866 plus NMN group had n = 5 at day 7, n = 5 at day 14, n = 6 at day 21, n = 5 at day 28, and n = 5 at day 35. For callus volume: Ctrl group had n = 6 at day 7, n = 11 at day 14, n = 7 at day 21, and n = 7 at day 28; NMN group had n = 6 at day 7, n = 10 at day 14, n = 11 at day 21, and n = 11 at day 28; FK866 group had n = 6 at day 7, n = 5 at day 14, n = 5 at day 21, and n = 5 at day 28; FK866 plus NMN group had n = 6 at day 7, n = 5 at day 14, n = 5 at day 21, and n = 5 at day 28). (I) Micro-CT analysis was employed to quantify bone volume in various experimental conditions: Ctrl (n = 5 at day 7, n = 11 at day 14, n = 7 at day 21), NMN-treated (n = 5 at day 7, n = 10 at day 14, n = 10 at day 21), FK866-treated (n = 6 at day 7, n = 5 at day 14, n = 5 at day 21), and FK866 plus NMN-treated (n = 6 at day 7, n = 5 at day 14, n = 5 at day 21). (J) Quantification of fracture callus parameters by Micro-CT measurements in the control and NMN groups (n = 7-11). (K) Biomechanical properties of uninjured or fractured femurs at DPF35 (n = 6-13). Data are presented as mean ± SEM; Statistical significance was determined by two-tailed unpaired Student's t test (C, J) or one-way ANOVA (H, I, K). (*p < 0.05, **p < 0.01, ***p < 0.001, ****p < 0.0001, n.s., not significant). In Fig. 1C, the difference between means in BV between the NMN group (n = 5) and the Ctrl group (n = 5) at DPF14 was 0.0169 (p = 0.0534, 95% CI [-0.0003, 0.0342]). The difference in the means of BV/TV between the NMN group (n = 5) and the Ctrl group (n = 5) at DPF14 was 12.25 (p = 0.0329, 95% CI [1.403, 23.10]). In Fig. 1J, CT-analyse; the difference in the means of trabecular number between the NMN group (n = 7) and the Ctrl group (n = 11) at DPF21 was 0.4155 (p = 0.0459, 95% CI [0.0086, 0.8224]). The difference in the means of BMD between the NMN group (n = 7) and the Ctrl group (n = 11) at DPF21 was 0.0372 (p = 0.0445, 95% CI [0.0010, 0.0733]). The difference in the means of structure model index between the NMN group (n = 7) and the Ctrl group (n = 11) at DPF21 was -0.3182 (p = 0.0109, 95% CI [-0.5526, -0.0838]).

To determine the effectiveness of NMN in facilitating bone injury repair in adult mice, we performed a drill hole injury model and administered NMN via drinking water for a period of 4 weeks (**Figure 1A**). For the 0.6-mm drilled defect, 3D reconstructed images from micro-CT scans revealed greater mineralized callus tissue at the injury site in the NMN-treated group compared with the control group at day 14 after surgery (**Figure 1B**). Meanwhile, NMN treatment showed a trend towards increased bone volume (BV) and significantly elevated the ratio of bone volume to total volume (BV/TV) at the injury site compared to the control group from day 7 to day 14 post-surgery (**Figure 1C**). These results suggest that NMN supplementation potentially expedites osteogenesis during the bone defect healing process.

To further explore the therapeutic role of NMN in callus formation, we subjected mice with transverse fractures of the femoral shaft at 2 months of age (**Figure 1D**). Different treatments were administered during the initial 6 days following the fracture, a critical period during which it influences the formation of the cartilaginous callus. Radiographic and micro-CT analyses demonstrated a significantly enhanced callus size in the NMN-treated group relative to the control group at 14 days post-fracture (DPF). Additionally, it was noted that the depletion of NAD using FK866, a specific inhibitor of the enzyme nicotinamide phosphoribosyltransferase (Nampt) resulted in a persistently diminished callus size. However, this reduction in callus size was counteracted by supplementation of NMN (**Figure 1E-1F**). Surprisingly, the administration of FK866 during the initial 6 days of post-fracture healing strikingly suppressed callus formation, with 28% of fractured femurs displaying no callus and resulting in fracture nonunion at 14-28 DPF (**Figure 1G**). The trends observed in the curves of callus volume and callus index were consistent with all these radiographic results (**Figure 1H**). NMN administration led to a modest increase in callus BV, yet notably mitigated the inhibited new bone formation provoked by FK866 (**Figure 1I**). Moreover, our micro-CT analysis validated that NMN treatment induced elevated trabecular number (Tb, N) and bone mineral density (BMD) within the callus from 14 to 21 DPF, alongside a reduction in the structure model index (SMI), with no discernible impact on trabecular separation (**Figure 1J**).

Biomechanical tests revealed a relative improvement in the maximum mechanical strength of NMN-treated mice at 5 weeks, but not significantly. In contrast, treatment with FK866 markedly compromised the mechanical strength of the fractured femur. However, co-administration of NMN with FK866 at the onset of fracture mitigated the adverse impact of NAD deficiency induced by FK866 on mechanical strength (**Figure 1K**). Altogether, these findings suggest that NMN potentially enhances the recovery process following bone injuries.

### NMN facilitates early cartilage formation after fracture

Given our observation of distinct healing endings following the administration of FK866, NMN and a combination of FK866 and NMN for 6 consecutive days post-fracture to influence NAD synthesis within callus, we proceeded to examine the callus formation of fractured femur at DPF7. The callus in NMN-treated group exhibited a preference for more cartilaginous and chondroid tissue rather than granulation tissue.

**Figure 2:**
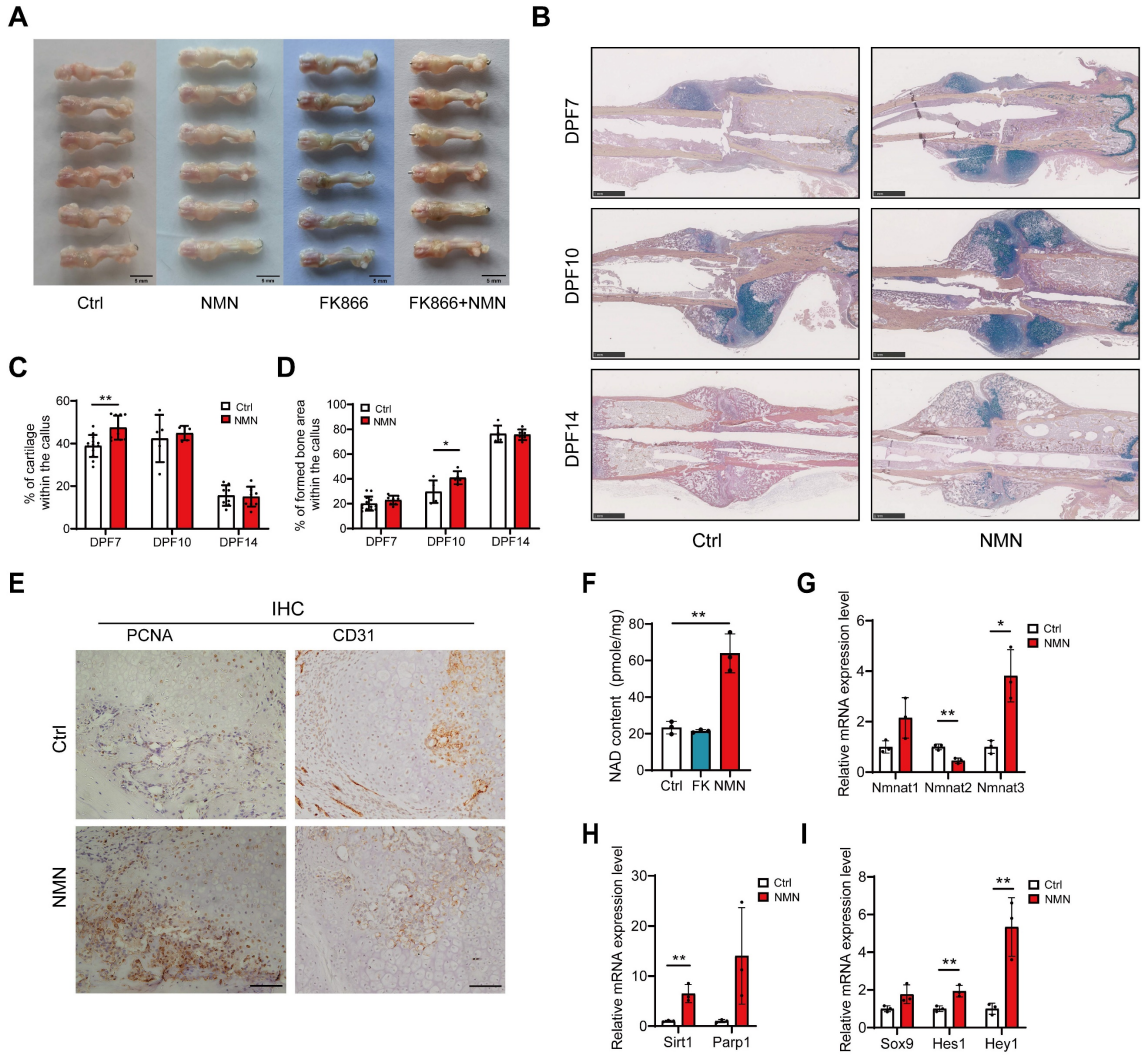
**NMN upregulates the content of NAD in the callus and promotes the formation of cartilaginous callus.** (A) Representative images of calluses treated with Saline, NMN, FK866, or a combination of FK866 and NMN at day 7 post-fracture. (B) Exemplary images of Movat's pentachrome staining depicting differences between control and NMN-treated callus over 14 days. (C, D) Quantitative assessment of fracture cartilage and newly generated bone proportion in the fracture calluses based on Movat's pentachrome staining (n = 5-13). (E) PCNA and CD31 expression were assessed by immunohistochemistry in the periosteal callus region. Scale bar, 100 μm. (F) Measurement of NAD content in fracture callus at post-fracture day 7 (n = 3). (G-I) Relative mRNA levels of Nmnat1, Nmnat2, Nmnat3, Sirt1, Parp1, Sox9, Hes1, and Hey1 were measured by qRT-PCR in callus samples (n = 3). Data are presented as mean ± SEM; Statistical significance was determined by two-tailed unpaired Student's t test (C, D, F, G, H, I). (*p < 0.05, **p < 0.01).

Additionally, the significantly smaller callus observed in the FK866 group was mitigated by additional NMN administration (**Figure 2A**). To investigate the effect of NMN administration on the early callus composition, we performed histomorphometry analysis on the sections of fractured callus stained with Movat's pentachrome at 7, 10, and 14 DPF (**Figure 2B**). The proportion of cartilage, derived from various types of chondroprogenitor cells, was evaluated, showing that the NMN group exhibited an average cartilage content of 47.5% at DPF7, markedly higher than the 38.9% observed in the control group (**Figure 2C**). The discrepancy in the proportion of cartilage between NMN-treated and control calluses was minimal at DPF10 and DPF14. However, histological analysis confirmed a more pronounced formation and mineralization of new bone in NMN group compared to the control group at DPF10, showing a 38% increase in NMN group (**Figure 2D**). Therefore, these data demonstrate that NMN can contribute to the formation of cartilaginous tissue and the subsequent transformation of new cancellous bone tissue, thereby accelerating fracture healing.

Furthermore, compared to control group, our histological results showed the proliferating cell nuclear antigen (PCNA) positive cells accumulated adjacent to the periosteum but far away from the fracture site at DPF7 with NMN treatment (**Figure 2E,** left panel), suggesting that NMN enhances cell proliferation in the periosteum region. Conversely, no significant difference in the number of PCNA-positive cells in external soft tissue was observed between NMN-treated and control mice. Chondrocytes were labeled using Movat staining and immunohistochemical staining for the chondrocyte-specific marker type II collagen (**Figure S1B**). In both NMN and control groups, few PCNA expression was observed in chondrocytes, which indicates that the predominant cell population undergoing proliferation in the early callus is not chondrocytes. Immunohistochemical staining of CD31, a vascular endothelial marker, revealed no difference in the callus between the NMN and control groups at DPF10 (**Figure 2E,** right panel).

Previous studies have demonstrated that administration of NMN either by intraperitoneal injection or oral gavage can rapidly transport NMN into systemic circulation, distributing it as NAD intermediates to multiple organs and tissues, thereby promoting NAD synthesis 19, 28. To explore whether early short-term administration of FK866 or NMN affects the initial formation of cartilaginous callus by influencing callus NAD levels, we measured the NAD content and related mRNA expression in callus at DPF7. As expected, the NAD content in the callus of the NMN group was approximately 2.5 times higher than that of the control group 24 hours after the last 500 mg/kg NMN injection. However, FK866 did not significantly reduce the total amount of NAD in callus (**Figure 2F**). NAD levels in the callus declined sharply within 12 hours after FK866 administration and rebounded over 24 hours (data not shown), whereas the callus in the FK866 group was collected more than 30 hours after the last administration, and NAD levels in the callus recovered. Another possible reason is that even though FK866 blocks the Nampt-mediated NAD salvage pathway, certain compensation from the *de novo* biosynthesis or Preiss-Handler pathway may activate.

The NMN adenylyltransferase (NMNAT), including NMNAT1, NMNAT2, and NMNAT3, are distributed across different cellular compartments, namely the nucleus, Golgi surface, and mitochondria, and are responsible for NAD biosynthesis 28. qRT-PCR analysis showed increased mRNA levels of NMNAT1 and NMNAT3, with a significant decrease in NMNAT2 mRNA levels (**Figure 2G**), suggesting NAD is preferentially synthesized in mitochondria and nuclei. The expression of NAD+-dependent enzymes like Sirt1 and Parp1, crucial for DNA damage repair, cell proliferation and circadian rhythms, increased in the NMN-treated group (**Figure 2H**). Besides, Sox9, a key chondrogenic differentiation marker, along with Hes1 and Hey1, downstream effectors of notch signaling, were significantly up-regulated after NMN treatment (**Figure 2I**). We further confirmed by western blotting that NMN upregulates Hey1 and Hes1 before the cartilaginous callus formation (**Figure S2C**). Taken together, these results suggest that NMN promotes the formation of cartilage callus by increasing the NAD content in the callus.

### NMN stimulates the proliferation of heterogeneous stem cells within the callus

The elevation in cartilage composition within the callus following NMN administration prompted us to investigate which cell types mainly respond to NMN, namely, the chondrocytes themselves or their progenitor cells. We preliminarily established a long-term NMN administration group by administering NMN from 0 to 14 DPF to cover the entire callus formation and maintenance period. However, the final results showed that the callus size and ultimate mechanical strength were not better than those of the early short-term (from 0 to 6 DPF) administration group (**Figure S1A, D, E, F**). Based on this finding, we speculated that NMN predominately affects cartilage progenitor cells, including SSCs and mesenchymal stem cells resident in the medullary cavity and periosteum, rather than chondrocytes.

**Figure 3:**
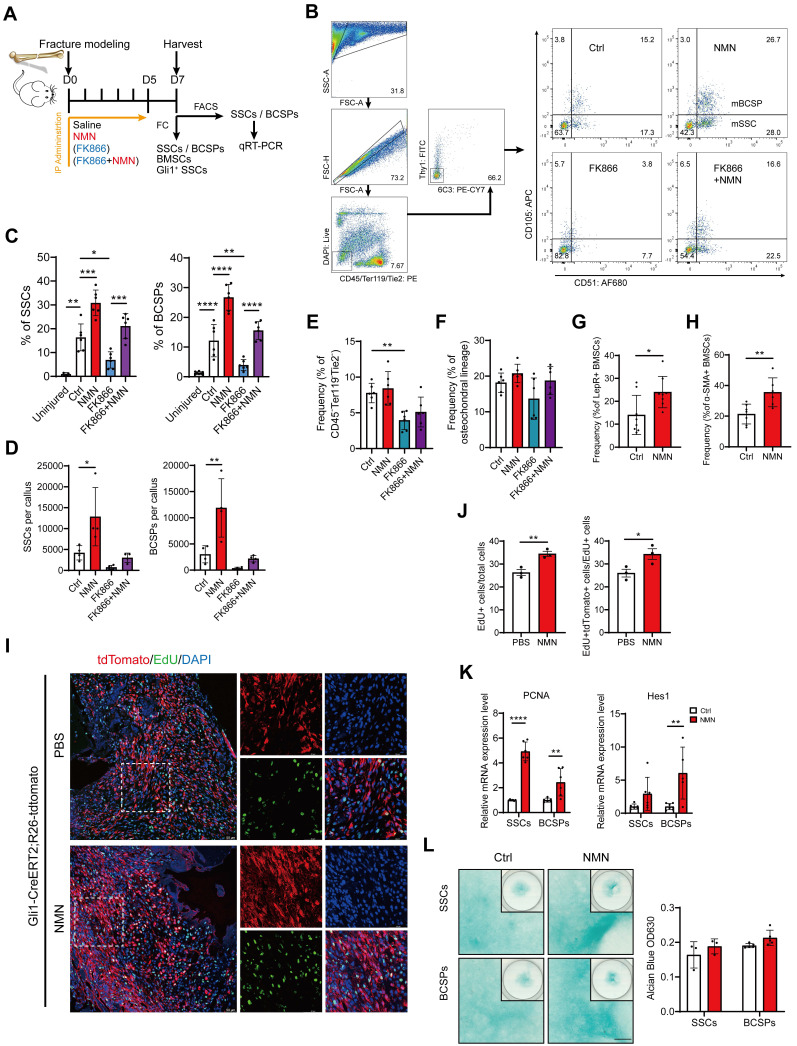
**NMN induces injury-activated stem cell expansion during skeletal regeneration.** (A) Schematic of the experimental design. NMN, FK866, and FK866 plus NMN were administered after fracture, and callus samples were collected at DPF7 for flow cytometric and qRT-PCR analysis. (B) Representative flow cytometry plots identifying SSCs and BCSPs from callus tissue in all treatment groups. (C) Quantification of SSC and BCSP frequency within the callus or uninjured femur (n = 6-8). (D) Quantification of SSC and BCSP numbers within the treated callus (n = 4). (E) Flow cytometric analysis of CD45-Ter119-Tie2- cells within callus (n = 6). (F) Quantification of osteochondral lineage cell frequency (n = 3-6). (G, H) Expansion of LepR+/α-SMA+ BMSCs after injury and their flow cytometric analysis. (n = 6-8). (I) Representative immunofluorescence images showing increased EdU labeling in Gli1+ periosteal SSCs from Gli1 creERT2; tdTomato mice 1 week after fracture following NMN supplementation compared to the control group (n = 3-5). Scale bar, 50 μm. (J) Quantification of the percentage of EdU+ cells and the percentage of proliferative Gli1+ cells within callus (n = 3). (K) qRT-PCR analysis of Hes1 and PCNA expression in FACS-purified SSCs and BCSPs (n = 5-7). (L) Left: Alcian blue staining of SSCs and BCSPs demonstrating chondrogenic ability in the control and NMN groups. Right: Quantification of chondrogenesis in each group (n = 3-5). Data are presented as mean ± SEM; Statistical significance was determined by two-tailed unpaired Student's t test (G, H, J, K, L) or one-way ANOVA (C, D, E, F). (*p < 0.05, **p < 0.01, ***p < 0.001, ****p < 0.0001).

Considering the involvement of SSCs in the initial repair process of bone injuries, we further examined NMN's effect on the regenerative capabilities of stem cells within the callus (**Figure 3A**). The cell population CD45-Ter119-Tie2-6C3-Thy1-CD105+CD51- was characterized as SSCs and CD45-Ter119-Tie2-6C3-Thy1-CD105+CD51+ was characterized as BCSPs. We discovered that NMN supplementation significantly enhances the injury-activated proliferation of SSCs and BCSPs at DPF7. However, the frequency and quantity of SSCs and BCSPs sharply decreased in the FK866 group, compared to control, but were effectively restored in the FK866 plus NMN group (**Figure 3B-3E**). Nonetheless, no notable variance was observed in the frequency of osteochondral lineage cells derived from BCSPs (**Figure 3F**). These findings align with our previous radiographic and micro-CT analyses of calluses, indicating a potential intrinsic correlation between the initial expansion of SSCs during fracture onset and the subsequent cartilaginous callus formation. To verify if NMN could induce the expansion of stem cells beyond SSCs/BCSPs, we conducted flow cytometric analysis on Lin- (CD45-Ter119-CD31-) LepR^+^ BMSCs and α-SMA^+^ BMSCs. As expected, the frequency of these BMSC subpopulations within the callus at DPF7 increased in the NMN group compared to the control (**Figure 3G-3H**).

Gli1^+^ periosteal SSCs have recently been reported to predominantly contribute to cartilaginous callus formation after fracture repair 29. The proliferative capacity of these cells was subsequently evaluated in the NMN and control groups post-surgery, utilizing Gli1 lineage tracer mice with EdU labeling. Our results showed a higher percentage of EdU labeled proliferative Gli1^+^ SSCs adjacent to the fracture site in the NMN group (**Figure 3I**). At DPF7, the NMN administration treatment increased the proportion of EdU^+^ cells within the callus, as well as the proportion of EdU^+^ Gli1^+^ cells within all EdU^+^ cells (**Figure 3J**). To observe the effect of NMN on other stem cells *in vivo*, FACS-isolated SSCs and BCSPs were collected for qRT-PCR analysis. NMN administration resulted in a significant upregulation of PCNA and Hes1 expression in both SSCs and BCSPs (**Figure 3K**), suggesting a potential link between NMN-induced stem cell expansion within the callus and the activation of the Notch signaling pathway. Collectively, these findings elucidated that NMN has a significant proliferative effect on stem cells in the callus.

We further investigated the impact of NMN on the chondrogenic differentiation of SSCs and BCSPs. Fluorescence-activated cell sorting (FACS)-purified SSCs and BCSPs were cultured in a micromass pattern with or without NMN supplementation for 20 days after seeding. Subsequently, they were stained with Alcian blue, as shown in **Figure 3L**. No significant differences were observed between NMN and the control group on SSCs/BCSPs' chondrogenesis, regardless of whether SSCs were seeded in high-density micromass cultures or in monolayer cultures (data not shown). Thus, NMN did not possess a significant capacity to enhance chondrogenic differentiation of SSCs.

### The pro-proliferative effect of NMN on SSCs depends on macrophages *in vivo*

**Figure 4:**
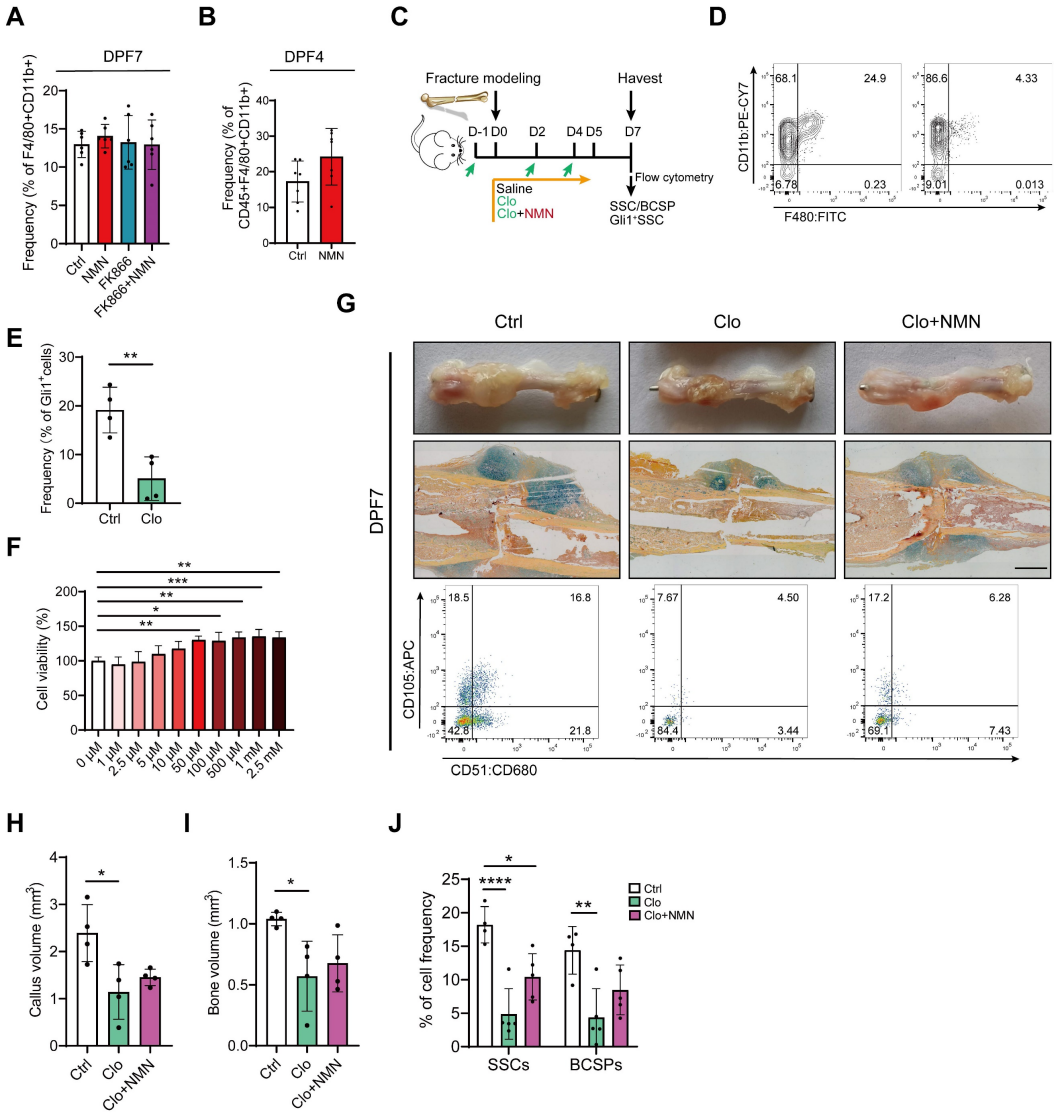
**Macrophage clearance impaired the stimulatory effect of NMN on stem cell proliferation in the callus.** (A) Quantification of macrophage frequency within the callus at DPF7 (n = 6). (B) Quantification of macrophage frequency within the callus at DPF4 (n = 7). (C) Schematic timeline of macrophage depletion assays. (D) Flow cytometry confirmed the efficacy of macrophage clearance. (E) Prevalence of Gli1+ SSCs and their progeny cells after surgery in Gli1 creERT2; tdTomato mice (n = 4). (F) Cell viability assay of macrophages after treatment with different concentrations of NMN for 24 hours using the CCK-8 assay (n = 6). (G) Representative photographs, Movat's pentachrome staining images, and SSCs flow plot of the callus at DPF7. (H, I) Callus volume and bone volume of the control group, chlorophosphate liposomes alone group, and chlorophosphate liposomes plus NMN treatment group (n = 4). (J) Frequency of SSCs and BCSPs within the callus from three groups (n = 4-5). Data are presented as mean ± SEM; Statistical significance was determined by two-tailed unpaired Student's t test (B, E) or one-way ANOVA (A, F, H, I, J). (*p < 0.05, **p < 0.01, ****p < 0.0001).

At the onset of fracture, during the inflammatory phase of hematoma formation, various types of immune cells, especially macrophages, play crucial roles in interacting with stem cells 30. Peter Currie *et al.* discovered that a specific subset of macrophage populations can accelerate injury repair soon after zebrafish muscle injury by stimulating the proliferation of neighboring stem cells 31. This finding inspired us to investigate the impact of macrophages on early bone fracture healing. Indeed, it remains uncertain whether NMN could induce alterations in the polarization or function of macrophages within the early callus after bone fracture, thereby influencing the proliferation of stem cells. We found that the frequency of macrophages did not change in every experimental group compared to the control at DPF7 (**Figure 4A**). With the rapid proliferation of SSCs initiated between the fourth and seventh day after fracture, there was an increase in macrophage abundance during this period. Although NMN may influence macrophage numbers to some extent at DPF4, the effect was not statistically significant (**Figure 4B**). We observed that BMSCs were more susceptible to FK866 toxicity compared to macrophages *in vitro* (**Figure S2C**), as determined by CCK-8 toxicity testing, indicating the primary mechanism by which FK866 inhibits callus formation may be likely through its hindrance of stem cell proliferation. Previous *in vitro* studies have confirmed that the polarization of macrophages is closely linked to the demand for NAD 32, 33. To investigate this concept, macrophages were treated with or without FK866 and NMN during the induction of M1 and M2 polarization, respectively. qRT-PCR results showed that NMN did not affect the M2 polarization of macrophages, while FK866 tended to inhibit M1 polarization (**Figure S2A-B**). Collectively, NMN exhibits a constrained effect on macrophage function.

While NMN itself does not influence macrophage function, there is a suspicion that the regulation of stem cells by NMN may be dependent on the presence and activity of macrophages. Next, we employed chlorophosphate liposomes (Clo) to deplete macrophages during the initial days of bone fracture and investigated whether NMN's impact on SSC proliferation was disrupted *in vivo* (**Figure 4C**). The average clearance efficiency of Clo on macrophages within the callus at DPF7 was approximately 80%, as confirmed by flow cytometry (**Figure 4D**). The ablation of macrophages compromised Gli1+ periosteal SSCs and their descendant cells (**Figure 4E**). To further assess the activity of NMN in the proliferation of macrophages, CCK-8 assays were used to detect the viability of macrophages treated with various concentrations of NMN *in vitro*. No toxic effects were observed on macrophages at concentrations below 2.5 mM of NMN, and its impact on their proliferative activity was modest (**Figure 4F**). Morphological and histological observations indicated that the callus at DPF7 treated with Clo was notably smaller than the control and not ameliorated by NMN supplementation (**Figure 4G**). Further, micro-CT analysis demonstrated a significant reduction in callus volume and bone volume at DPF7 following macrophage depletion (**Figure 4H-I**). Likewise, we observed a significant decrease in the frequency of both SSCs and BCSPs in the callus of Clo-treated group, with a non-significant increase in the Clo plus NMN group compared to the Clo group (**Figure 4G, J**). Additionally, the implantation of unpolarized macrophages immediately after fracture markedly increased macrophage abundance within the callus at DPF7, but did not contribute to SSCs or BCSPs expansion (**Figure S2D-E**). These findings underscore the indispensable role of macrophages in facilitating the expansion of SSCs that initiate bone injury repair, while also revealing NMN's limited effect on macrophage proliferation and polarization during early fracture callus formation. However, a more profound correlation between macrophages and SSCs may need to be explored.

### NMN promotes BMSCs and SSCs' proliferation *in vitro*

**Figure 5:**
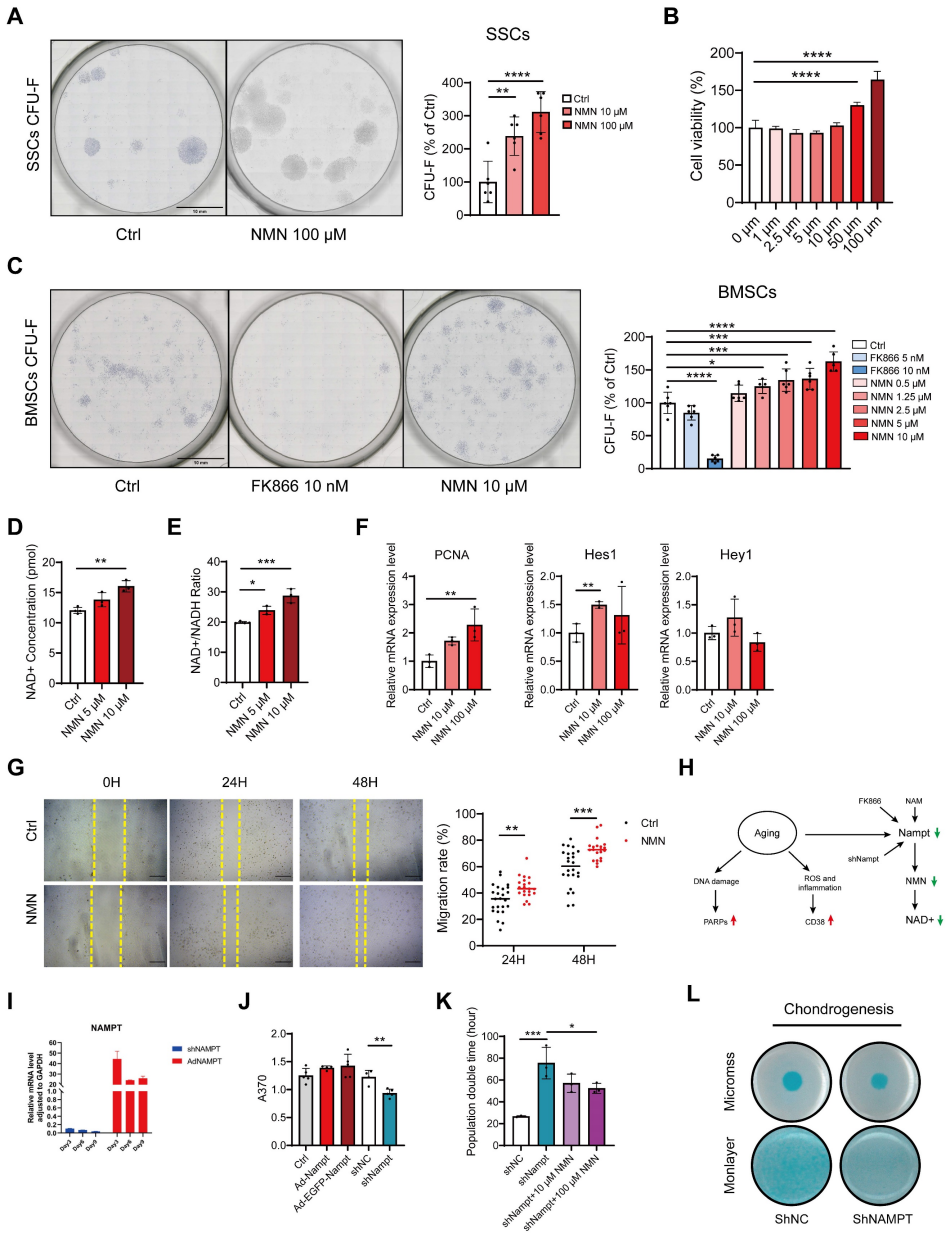
**NAD is responsible for the regulation of stem cell expansion *in vitro*.** (A) Representative images depict CFU-Fs stained with toluidine blue on SSCs and the quantity of SSC colonies following NMN treatment (n = 6). (B) Cell viability assay of SSCs following 24-hour treatment with various concentrations of NMN, assessed by CCK-8 assay (n = 6). (C) Representative images depict CFU-F colonies generated by BMSCs and quantification of CFU-F numbers compared to control (n = 5-6). (D, E) Increased NAD+ concentration and NAD+ / NADH ratio were observed with NMN supplementation (n = 3-4). (F) Relative gene expression levels of PCNA, Hes1, and Hey1 in NMN-treated BMSCs compared to controls (n = 3). (G) Representative images depicting wound healing of BMSCs treated with 10 μM NMN at 0, 24, and 48 hours post-treatment. Migration rate of BMSCs (n = 7-8). (H) Schematic illustrating the age-related decrease in NAD levels attributed to reduced Nampt expression in the NAD salvage pathway. (I) qRT-PCR validated the efficiency of knockdown and overexpression of Nampt in constructed C3H10T1/2 cells. (J) EdU assay revealed that knockdown of Nampt impeded C3H10T1/2 cell proliferation (n = 4-5). (K) Population doubling times assay was conducted on Nampt-knockdown C3H10T1/2 cells with or without NMN treatment (n = 3). (L) C3H10T1/2 cells in monolayer or micromass were cultured for 21 days before staining with Alcian blue. Data are presented as mean ± SEM; Statistical significance was determined by two-tailed unpaired Student's t test (F, G, J, K) or one-way ANOVA (A, B, C, D, E,). (*p < 0.05, **p < 0.01, ***p < 0.001, ****p < 0.0001).

To further validate if NMN could induce stem cell expansion independently of macrophages *in vitro*, we conducted a colony-forming unit fibroblasts (CFU-F) assay. Toluidine blue staining of FACS-sorted SSCs with continuous NMN supplementation showed the average number of colonies in the NMN 10 μM group and 100 μM group increased by 126% and 195%, respectively, compared to the control group (**Figure 5A**). The results of the CCK-8 assay showed that NMN increased the viability of FACS-purified SSCs, reaching a maximum at 100 μM (**Figure 5B**). Similar CFU-F results were observed in a CFU-F assay performed on BMSCs, demonstrating that NMN led to a dose-dependent increase in CFU-Fs, while few CFU-Fs were observed in the FK866-treated group (**Figure 5C**). In BMSCs culture, the cellular NAD+ and NAD+/NADH ratios, which are intricately linked to cellular function, are elevated by NMN (**Figure 5D-E**). These results indicate that the content of NAD in stem cells was positively correlated with their proliferative ability *in vitro*. Analysis of BMSCs at the transcriptional level revealed an increase in PCNA and Hes1 expression following NMN treatment. However, no statistically significant differences were found in the expression levels of Hey1 (**Figure 5F**). No enhancement of the Sirts family transcripts of BMSCs or SSCs was observed with NMN administration *in vitro* (**Figure S3A-B**), and the mtDNA copy number remained unaffected (**Figure S3C**). Taken together, we have directly demonstrated that NMN stimulates the proliferation of SSCs and BMSCs* in vitro*. We also explored NMN's effects on BMSCs migration by conducting a wound scratch test. The results revealed that NMN at a concentration of 10 μM increased the migration ratio of BMSCs at both 24 hours and 48 hours (**Figure 5G**).

It was also demonstrated that BMSCs in both artificial induction (ex. ovariectomized model) and natural aging animals lead to a decline in Nampt protein expression and NAD levels, a phenomenon also mimicked by FK866, resulting in a series of aging traits (**Figure 5H**). To explore the potential impact of endogenous Nampt activation on stem cell proliferation, lentiviral vectors were employed to knock down and overexpress Nampt in the mouse mesenchymal stem cell line C3H10T1/2. Either knockdown or overexpression efficiency of the constructed C3H10T1/2 cell lines was examined by qRT-PCR (**Figure 5I**). EdU assay revealed that the proliferation capacity was attenuated in Nampt-deficient C3H10T1/2 cells, while it was slightly increased by overexpression of Nampt (**Figure 5J**). Additionally, Nampt deficiency extended the proliferation period of C3H10T1/2 cells which was ameliorated by NMN supplementation (**Figure 5K**). Furthermore, Alican blue staining revealed blunted chondrogenesis in Nampt-deficient C3H10T1/2 cells compared to control C3H10T1/2 cells (**Figure 5L**). These findings underscore the importance of Nampt in stem cell self-renewal and chondrogenic differentiation.

## Discussion

NMN, as a NAD-increasing agent, can significantly alleviate the decline in NAD levels associated with physiological aging and related diseases. NMN, NR, and other NAD intermediates have been shown to effectively ameliorate aging-induced bone loss by increasing circulatory NAD content 19, 26, 27, which highlights the importance of NAD in maintaining skeletal homeostasis and consequently reduce the risk of fractures. Even under extreme conditions such as radiation and simulated microgravity, NMN can promote stem cell expansion and reverse mitochondrial damage in osteoblasts, thus partially restoring bone mass 27, 34. Our study demonstrates that NMN administration enhances the healing of bone defects in adult mice, indicating a potential therapeutic role for NMN in promoting bone regeneration. This finding aligns with recent research where an NMN-based hyaluronic acid methacryloyl hybrid hydrogel scaffold was shown to expedite the repair of cranial bone defects in rats 35. Zufu Lu *et al.* found that long-term administration of NMN could increase the bridging rate of fractured callus in ovariectomized mice 36, but a further demonstration of the exact efficacy of NMN on fracture is still needed. Using a transverse femoral fracture model, we systematically investigated the impact of NAD on the initial phases of fracture healing in healthy male mice, meticulously analyzing phenotypic variations across groups treated with saline, FK866, NMN, and a combination of FK866 and NMN. The longitudinal study reveals that NAD levels during the early stages of cartilaginous callus formation significantly affect overall fracture repair. In contrast, early short-term supplementation with FK866 suppresses preliminary cartilaginous callus formation and significantly increases the likelihood of nonunion at the end of the healing process. This finding coincides with the results reported by Boer Li *et al.*, demonstrating that the continuous administration of FK866 throughout the fracture healing process results in significantly compromised fracture repair. From the standpoint of energy metabolism, FK866 adversely affects mitochondrial oxidative phosphorylation within stem cells, consequently impeding the normal progression of fracture healing 37. Interestingly, this abnormal phenotype can be counteracted by NMN treatment. Additionally, the total amount of NAD in the callus significantly increased after NMN administration, with NAD preferentially synthesized in the nucleus and mitochondria of callus cells. Furthermore, NMN enhanced the cartilaginous proportion within the callus, leading to more newly formed woven bone after 14DPF. These results support that a sufficient NAD pool during the initial stages of fracture healing may ensure postnatal bone healing after injury.

Stem cells derived from diverse origins not only uphold skeletal homeostasis in mature individuals but also intricately contribute to the reparative mechanisms involved in bone injury. Upon proliferating sufficiently in the early stages of injury, wound-activated stem cells differentiate into chondrocytes or osteoblasts to contribute to the subsequent formation of the fractured callus, during which they transition their energy metabolism from glycolysis to oxidative phosphorylation. There is considerable evidence linking NAD+ metabolism with the regulation of cell fate. Studies have shown that NAD+ manipulates osteogenic differentiation of BMSC through oxidative phosphorylation. Inhibition of intracellular NAD levels by FK866 significantly attenuated osteogenesis, a process that can be reversed by supplementation with NMN 37. These findings led us to re-examine the biological roles of NMN in stem cell proliferation, rather than osteogenesis, during callus formation. We confirmed that NMN stimulates SSCs proliferation within callus, rather than directly affecting chondrocytes. Both LepR^+^ and α-SMA^+^ BMSCs within the callus have also been shown to expand in response to NMN administration, supporting the perspective that increasing NAD levels by precursors can induce stem cell proliferation in injured tissues. *In vitro* experiments further corroborate this phenomenon, showing that NMN can enhance stem cell viability, expansion, and migration capacity. Emerging evidence substantiates the role of NMN or NR in promoting stem cell proliferation, activating hematopoiesis through modulation of cellular mitochondrial quality 38, and fostering the proliferation of vascular smooth muscle cells 39. Notch signaling has been demonstrated to regulate stem cell properties in skeletal development and regeneration 40, 41. We also noticed that Hes1 was upregulated in FACS-sorted SSCs and BCSPs after NMN supplementation. These observations suggest that the mechanisms by which NMN promotes stem cell proliferation may be distinct from its effects on mitochondrial function or sirtuin activity. Further investigation is required to elucidate the underlying pathways through which NMN exerts its proliferative effects on stem cells during tissue repair and regeneration. It is worth noting that stem cell transplantation holds significant promise in bone injury repair research. BMSCs are extensively studied for their role in promoting fracture healing and bone repair, owing to their capacity for self-renewal and multi-directional differentiation 42, 43. The effective drug delivery systems tailored for these NAD intermediates, whether utilized independently or in conjunction with stem cell therapies, hold substantial practical significance, particularly for the treatment of bone injuries in elderly patients.

Macrophages have recently been reported to participate in the crosstalk and interaction with stem cells during successful wound healing 44, 45. Our study finds that NMN has a limited effect on the cell viability of macrophages compared to BMSCs and does not directly promote the expansion and polarization of macrophages. Although NAD+ salvage has been confirmed to dominate macrophage metabolic shifts and polarization *in vitro* 32, 33, the response of macrophages to NMN and their potential change in cellular identity and function during the initial stages of fracture repair remains uncertain. Interestingly, we observed that the ablation of macrophages by Clo reduced the number of proliferating SSCs, BCSPs and Gli1+ lineage cells within the callus. This suggests that macrophage clearance seriously impinges on the recruitment of stem cells or blocks the initiation of stem cell proliferation. While exogenous NMN was able to substantially reverse the detrimental effects of FK866 on callus formation, it failed to rescue the depleted callus in the Clo-treated group. Through cytotoxicity tests, we found that stem cells exhibit greater susceptibility to FK866 compared to macrophages, suggesting that the inhibition of callus formation and subsequent delayed or nonunion is primarily attributed to FK866's impairment of stem cells rather than macrophages.

Moreover, treatment with Clo plus NMN showed a slight increase in stem cell frequency, though not statistically significant, indicating a limited regenerative capacity of NMN on SSCs under macrophage-depleted conditions. Given the direct influence of macrophage-stem cell interactions on initial tissue repair, we probed post-fracture transplantation of M0 macrophages. The results revealed that while exogenous macrophages contributed to callus formation at DPF7, they did not further enhance stem cell proliferation. Due to the high heterogeneity and plasticity of the macrophage population, it remains uncertain whether transplanting macrophages in M1 or M2 polarized states would yield divergent outcomes. This finding highlights the potential indispensability of macrophages for the recruitment and proliferation of stem cells following a fracture. In a zebrafish muscle injury model, Nampt secretion from the ecological niche of tissue-resident macrophages has been demonstrated to act through C-C motif chemokine receptor 5 (Ccr5) expressed within skeletal muscle stem cells, thereby promoting their proliferation. Furthermore, the application of a hydrogel patch containing Nampt to a mouse model of muscle injury similarly stimulated the repair of muscle damage in mice 31. This encouraging result emboldens further exploration into elucidating the precise role of Nampt or NAD in orchestrating the intricate interplay between macrophages and skeletal stem cells in our subsequent murine fracture models.

## Conclusion

In summary, the pivotal discovery of this study is that the early short-term administration of the NAD+ intermediate NMN induces injury-activated SSCs to acquire regenerative potential and facilitates subsequent endochondral ossification by forming a larger cartilaginous callus in a mouse fracture model. These findings provide valuable insights into the potential therapeutic value of NAD+ in tissue repair processes. Given these promising results, further effort is suggested to explore the therapeutic potential of NMN for the prevention and treatment of osteoporotic fractures, offering a novel avenue for improving bone health and recovery.

## Supplementary Material

Supplementary figures and table.
