# Supplementary material for: Nicotinamide mononucleotide enhances fracture healing by promoting skeletal stem cell proliferation
Source: Theranostics. 2024 Sep 16;14(15):5999–6015. doi: 10.7150/thno.98149 (PMC11426247; doi:10.7150/thno.98149)
Supplement: Supplementary file 1 — Supplementary figures and table. [file thnov14p5999s1.pdf]

Supplementary materials for

Nicotinamide mononucleotide enhances fracture healing by promoting skeletal stem cell proliferation

Yitian Shi[1, †], Jiayin Peng[3 †], Mengfan Liu[1], Xiling Qi[1], Siyu Li[1], Qiangqiang Li[4], Qing Jiang[4], Liming Zheng[5], Jiankun Xu[6], Yun Zhao[3 *], Yifeng Zhang[1, 2 *]

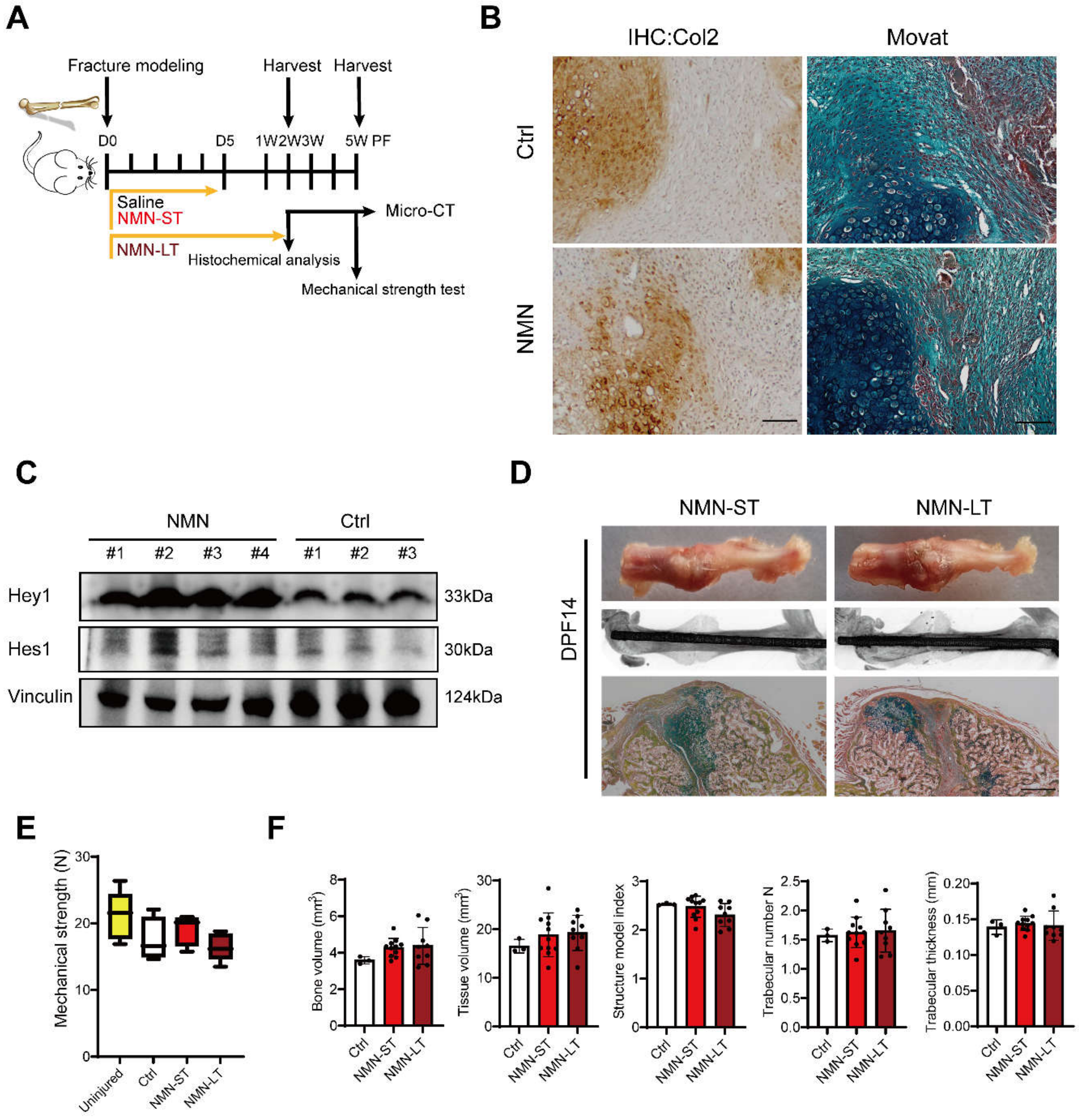

Figure S1. Comparison between long-term and short-term NMN dosing groups for fracture healing.

(A) Schematic illustrating the short-term and long-term administration of NMN, followed by assessment. NMN administration commenced 6 days after fracture in the short-term group and 14 days after fracture in the long-term group.

(B) Characterization of chondrocytes within callus was performed using immunohistochemical staining for type II collagen and Movat staining. Scale bar: 100 μm.

(C) Western blot was used to detect the expression of Hey1 and Hes1 (n = 3-4).

(D) Representative photographs and radiographs of the fractured femur in NMN-ST and NMN-LT group at DPF14 (top and middle). Bottom, images of Movat's pentachrome staining.

(E) Biomechanical assessment of the fractured femur was conducted at DPF35 (n = 4-

6).
(F) Quantification of fracture callus parameters by Micro-CT measurements at DPF14 (n = 3-11).
Data are presented as mean ± SEM; Statistical significance was determined by one-way ANOVA (E, F).

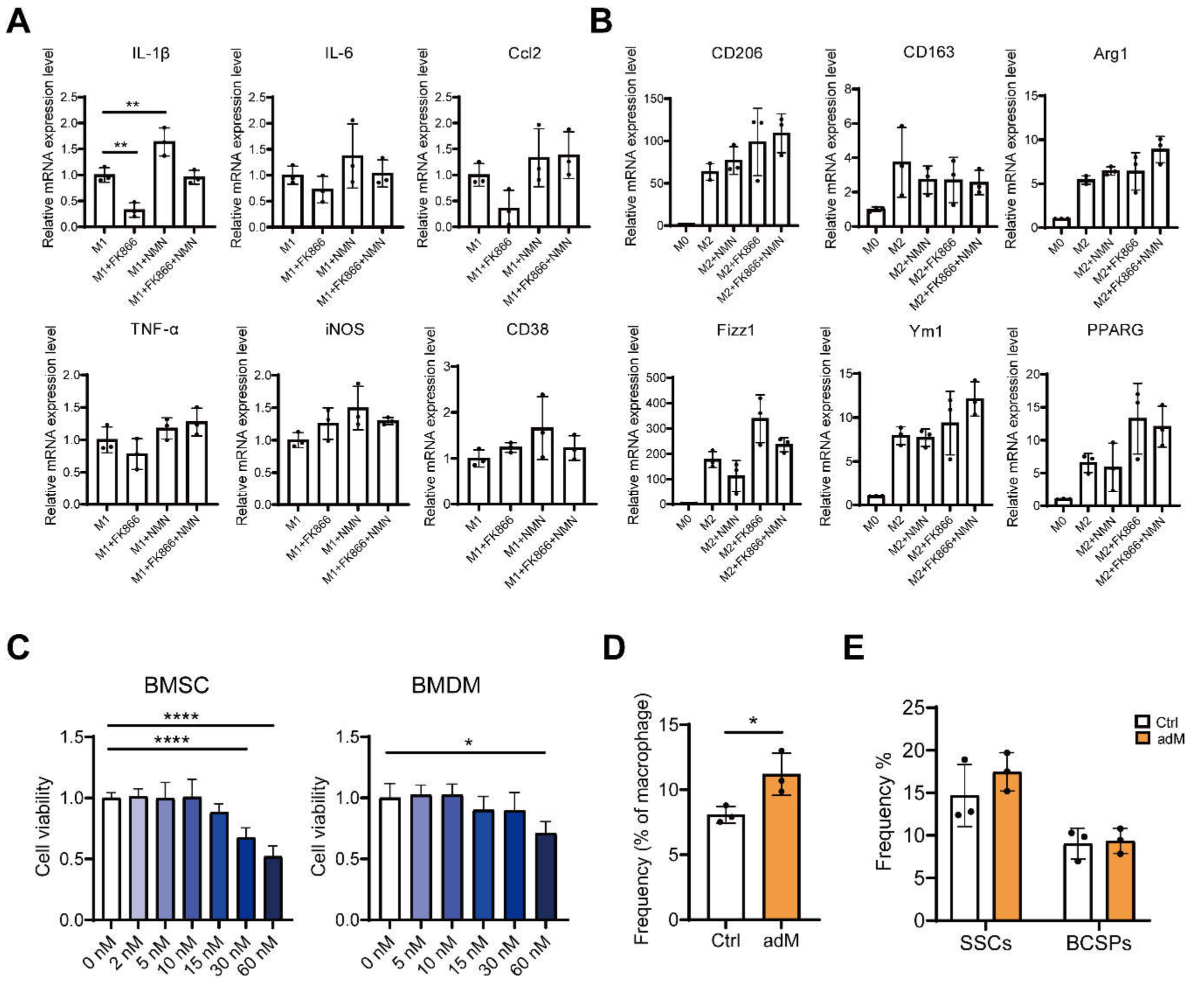

Figure S2. The effect of modulation of macrophage NAD levels on their polarization and activity *in vitro*; changes in the frequency of macrophages and skeletal stem cells in callus generated by the transplantation of macrophages at the time of fracture.

(A) qRT-PCR results show the effect of NMN or FK866 on M1 marker expression during macrophage M1 polarization (n = 3).

(B) qRT-PCR results show the effect of NMN or FK866 on M2 marker expression during macrophage M2 polarization (n = 3).

(C) The results of the CCK-8 toxicity assay showed that FK866 was more toxic to BMSCs than to macrophages (n = 5-6).

(D) Macrophage frequency within the callus was measured at DPF7 when macrophages were transplanted at the time of fracture (n = 3).

(E) Frequency of SSCs and BCSPs within the callus was measured at DPF7 when macrophages were transplanted at the time of fracture (n = 3).

Data are presented as mean ± SEM; Statistical significance was determined by two-tailed unpaired Student's t test (D, E) or one-way ANOVA (A, B, C).

(*$p < 0.05$, **$p < 0.01$, ****$p < 0.0001$).

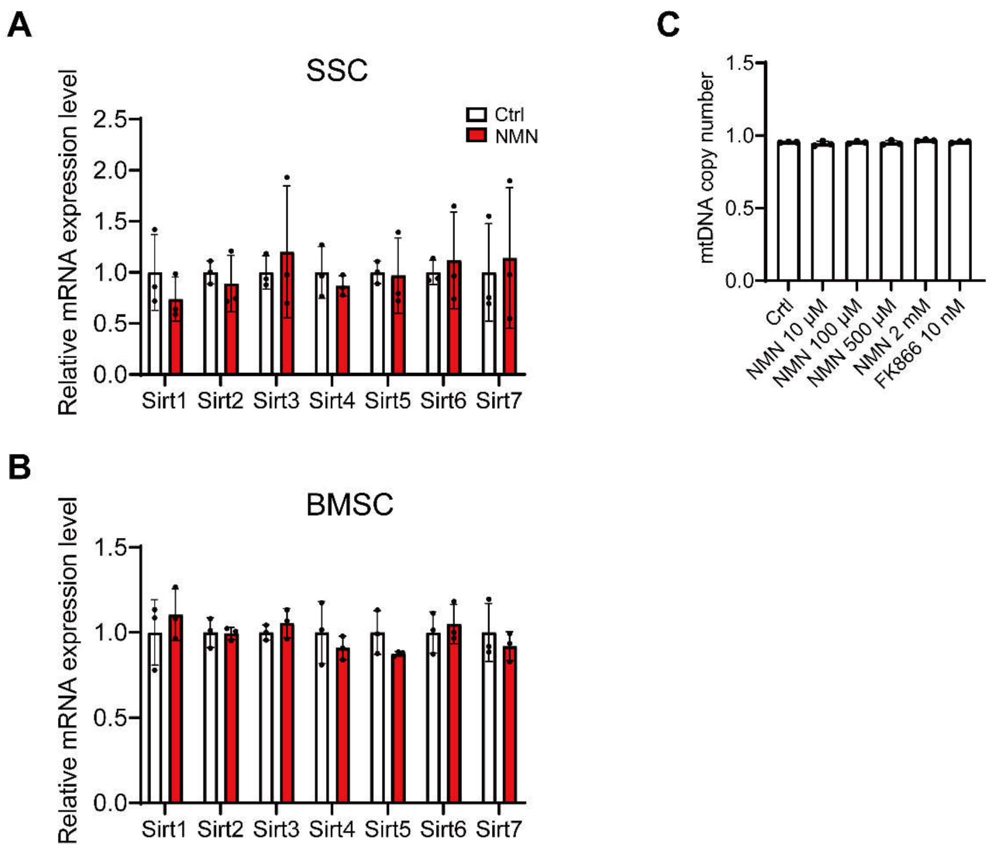

Figure S3. The effect of NMN on the transcript levels of the Sirtuin family and mitochondrial DNA copy number in stem cells.

(A) qRT-PCR results show that NMN does not alter the expression levels of the SIRT family in SSCs (n = 3).

(B) qRT-PCR results show that NMN does not alter the expression levels of the SIRT family in BMSCs (n = 3).

(C) qRT-PCR results show that NMN does not affect the copy number of mitochondrial DNA in BMSCs (n = 3).

Data are presented as mean ± SEM; Statistical significance was determined by two-tailed unpaired Student's t test (A, B) or one-way ANOVA (C).

Table S1: List of primer sequences used for qRT-PCR.

Primer Name	Sequence (5’-3’)
β-actin	FOR - AGCCTCGCCTTTGCCGA<br>REV - CTGGTGCCTGGGGCG
Gapdh	FOR - CTACACTGAGGACCAGGTTGTCT<br>REV - TTGTCATACCAGGAAATGAGCTT
Nampt	FOR - GCAGAAGCCGAGTTCAACATC<br>REV - TTTTCACGGCATTCAAAGTAGGA
Nmnat1	FOR - GAAATTGCTGTGTGGGGCAG<br>REV - CCACGATTTGCGTGATGTCC
Nmnat2	FOR - GATGTTCGAGAGAGCCAGGG<br>REV - AAGGCCCTGTTTTCCGTAGG
Nmnat3	FOR - AAGACACCATCAGCCTCTGC<br>REV - CCAAGCCGAACTTCTCCACT
Sirt1	FOR - AGTTCCAGCCGTCTCTGTGT<br>REV - GATCCTTTGGATTCCTGCAA
Sirt2	FOR - CAAGGAAAAGACAGGCCAGACGG<br>REV - CCTGACTGGGCATCTATGTTGGC
Sirt3	FOR - CCCTGTCTGTACTGGCGTTGTGA<br>REV - TCCATCCAGCTTGCCACGTTCC
Sirt4	FOR - TTCTCCTCTCACCAACCCAACCC<br>REV - TTCAGGCAAGCCAAATCGTCAG
Sirt5	FOR - ATGCGACCTCTCCTGATTGCTCC<br>REV - CCTCCCTCCGGTAGTGGTAAAAC
Sirt6	FOR - TGCAACCCACAAAACATGACCG<br>REV - GTATAGGGCTGTTGGGCTTGGAC
Sirt7	FOR - GAGCGAGGATCTGGTGACCGAG<br>REV - CAGGAGGTGCAGACTTCAATATACAT
Parp1	FOR - GGCAAGCACAGTGTCAAAGG<br>REV - TGTCGTTGACACCAGATGGG
PCNA	FOR - TGGAATCCCAGAACAGGAG<br>REV - TCAGAGCAAACGTTAGGTG
Sox9	FOR - AGCTCAACCAGACCCTGAGAA<br>REV - TCCCAGCAATCGTTACCTTC
Hes1	FOR - TGCCAGCTGATATAATGGAG<br>REV - CTTTGATGACTTTCTGTGCTC
Hey1	FOR - ACTACAGCTCCTCAGATAGTG<br>REV - AACTCAAGTTTCCATTCTCGTC
CD115	FOR - TGTGCAAGACCATGGTGAAT<br>REV - TTTTATCTGTGGGGGCTCTG
CD206	FOR - CAAGGAAGGTTGGCATTTGT

	REV - CCTTTCAGTCCTTTGCAAGC
CD163	FOR - TCAGCGACTTACAGTTTCCTC<br>REV - GCCTTTGAATCCATCTCTTG
Arg1	FOR - CTCCAAGCCAAAGTCCTTAGAG<br>REV - GGAGCTGTCATTAGGGACATCA
Fizz1	FOR - CCAATCCAGCTAACTATCCCTCC<br>REV - ACCCAGTAGCAGTCATCCCA
Ym1	FOR - CAGGTCTGGCAATTCTTCTGAA<br>REV - GTCTTGCTCATGTGTGTAAGTGA
IL10	FOR - CAGGGATCTTAGCTAACGGAAA<br>REV - GCTCAGTGAATAAATAGAATGGGAAC
iNOS	FOR - GGAGTGACGGCAAACATGACT<br>REV - TCGATGCACAACTGGGTGAAC
TNF-α	FOR - AGTGACAAGCCTGTAGCCC<br>REV - GAGGTTGACTTTCTCCTGGTAT
Ccl2	FOR - CCAGCAAGATGATCCCAATG<br>REV - TGGTTCCGATCCAGGTTTT
IL6	FOR - TGTATGAACAACGATGATGCACTT<br>REV - ACTCTGGCTTTGTCTTTCTTGTTATCT
IL1	FOR - CTGGTACATCAGCACCTCAC<br>REV - AGAAACAGTCCAGCCCATAC
PPARG	FOR - ACGATCTGCCTGAGGTCTGT<br>REV - CATCGAGGACATCCAAGACA
CD38	FOR - CGAAGGAGCTTCCAGTAACG<br>REV - TGGCAGGCCTGTAGTTATCC
36B4	FOR - ACTGGTCTAGGACCCGAGAAG<br>REV - TCAATGGTGCCTCTGGAGATT
Cytb	FOR - CCCACCCCATATTAAACCCG<br>REV - GAGGTATGAAGGAAAGGTATTAGGG
